# Cannabinoid Signaling and Autophagy in Oral Disease: Molecular Mechanisms and Therapeutic Implications

**DOI:** 10.3390/ijms27010525

**Published:** 2026-01-04

**Authors:** Undral Munkhsaikhan, Md Ataur Rahman, Alivia Shasteen, Karima Ait-Aissa, Amal M. Sahyoun, Rajat Das Gupta, Modar Kassan, Ehsanul Hoque Apu, Ammaar H. Abidi

**Affiliations:** 1 College of Dental Medicine, Lincoln Memorial University, 1705 St. Mary Street, Knoxville, TN 37917, USAehoqueapu@neomed.edu (E.H.A.); 2 Department of Oncology, Karmanos Cancer Institute, Wayne State University, Detroit, MI 48201, USA; rahman23@wayne.edu; 3 Department of Epidemiology and Biostatistics, Arnold School of Public Health, University of South Carolina, Columbia, SC 29208, USA; 4 Division of Epidemiology, Vanderbilt University Medical Center, Nashville, TN 37232, USA; 5 Department of Specialty Dentistry, Bitonte College of Dentistry, Northeast Ohio Medical University, Rootstown, OH 44272, USA; 6 Department of Biomedical Sciences, Northeast Ohio Medical University, Rootstown, OH 44272, USA

**Keywords:** cannabinoids, autophagy, oral cancer, periodontitis, CB1/CB2 receptors, mTOR signaling

## Abstract

Autophagy is a well-preserved biological mechanism that is essential for sustaining homeostasis by degradation and recycling damaged organelles, misfolded proteins, and other cytoplasmic detritus. Cannabinoid signaling has emerged as a prospective regulator of diverse cellular functions, including immunological modulation, oxidative stress response, apoptosis, and autophagy. Dysregulation of autophagy contributes to pathogenesis and treatment resistance of several oral diseases, including oral squamous cell carcinoma (OSCC), periodontitis, and gingival inflammation. This review delineates the molecular crosstalk between cannabinoid receptor type I (CB1) and type II (CB2) activation and autophagic pathways across oral tissues. Cannabinoids, including cannabidiol (CBD) and tetrahydrocannabinol (THC), modulate key regulators like mTOR, AMPK, and Beclin-1, thereby influencing autophagic flux, inflammation, and apoptosis. Experimental studies indicate that cannabinoids inhibit the PI3K/AKT/mTOR pathway, promote reactive oxygen species (ROS)-induced autophagy, and modulate cytokine secretion, mechanisms that underline their dual anti-inflammatory and anti-cancer capabilities. In addition, cannabinoid-induced autophagy has been shown to enhance stem cell survival and differentiation, offering promise for dental pulp regeneration. Despite these promising prospects, several challenges remain, including receptor selectivity, dose-dependent variability, limited oral bioavailability, and ongoing regulatory constraints. A deeper understanding of the context-dependent regulation of autophagy by cannabinoid signaling could pave the way for innovative therapeutic interventions in dentistry. Tailored cannabinoid-based formulations, engineered for receptor specificity, tissue selectivity, and optimized delivery, hold significant potential to revolutionize oral healthcare by modulating autophagy-related molecular pathways involved in disease resolution and tissue regeneration.

## 1. Introduction

Autophagy is a well-preserved catabolic mechanism activated under diverse cellular stress conditions to degrade and recycle damaged organelles, misfolded proteins, and other cytoplasmic components. By mitigating cellular damage, autophagy enhances survival during energy or food deprivation and protects against cytotoxic stressors [1]. Autophagy plays a central role in regulating cell survival, differentiation, and immune responses, while it is linked to multiple disorders, including cancer, neurodegeneration, and inflammatory disease [2]. In oral health, autophagy has emerged as a key regulator of disease progression and tissue remodeling [3]. Impairments in autophagic function are associated with periodontitis, oral mucositis, gingivitis, dental pulp necrosis, and oral squamous cell carcinoma (OSCC). Autophagy contributes to host defense by neutralizing damaged cellular components and limiting pro-inflammatory cytokine secretion [4]. During infection-induced inflammation, bacterial pathogens can trigger autophagy in host cells as a protective mechanism to eliminate intracellular microbes [5]. Understanding the upstream signaling systems that regulate autophagy in oral tissues is crucial for developing innovative treatment strategies.

Cannabinoids are a diverse class of bioactive lipophilic compounds derived from Cannabis sativa and other plant species, as well as synthesized endogenously and pharmacologically, and have attracted significant attention for their immunomodulatory, anti-inflammatory, antioxidant, and anticancer effects [6]. Among the several cannabinoids, cannabidiol (CBD) and Δ^9^-tetrahydrocannabinol (THC) are the most extensively studied [7]. Their biological actions are mediated primarily through cannabinoid receptors type I and type II (CB1 and CB2), G-protein–coupled receptors expressed in both central and peripheral tissues, including the oral mucosa, gingival epithelium, periodontal ligament, and dental pulp [8]. The activation of these receptors triggers intracellular signaling pathways that interact with essential autophagy regulators, such as mammalian Target of Rapamycin (mTOR), AMP-activated protein kinase (AMPK), Protein Kinase B (AKT), and Beclin-1.

Recent findings highlight the pivotal role of cannabinoid signaling in regulating autophagy in various cell types and tissues [9]. This connection is particularly critical in oral disease, where autophagy serves as a central homeostatic mechanism in maintaining epithelial integrity, modulating inflammatory responses, and controlling microbial balance within the oral cavity. Dysregulated autophagy in oral keratinocytes, fibroblasts, and immune cells contributes to chronic inflammation, impaired wound healing, and carcinogenesis, key features of conditions such as periodontitis, oral mucositis, and OSCC [10,11]. Mechanistic target of rapamycin (mTOR) is a crucial regulator of growth, anabolic metabolism, and aging, usually suppressing ULK1 (serine/threonine kinase). When cannabinoid receptors (CB1 and CB2) are activated, they inhibit mTOR signaling, which in turn activates the ULK1 complex. ULK1 plays a crucial role in initiating autophagy by phosphorylating Beclin-1, a core scaffold protein essential for the process. This phosphorylation activates the class III PI3K complex (also known as the Vps34 complex), which forms a phagophore that engulfs cellular debris and eventually forms an autophagosome [12]. Autophagosome maturation involves coordinated membrane expansion, cargo sequestration, and vesicle closure. A key step in this process is the conjugation of microtubule-associated protein light chain 3 (LC3) to phosphatidylethanolamine, forming LC3-II. This lipidated form of LC3 stably associates with the autophagosomal membrane and facilitates membrane expansion, cargo recognition through LC3-interacting region (LIR)-containing adaptor proteins, and autophagosome closure [5]. The completed autophagosome then fuses with a lysosome to form an autolysosome, where lysosomal hydrolases degrade the sequestered materials. The resulting macromolecular constituents, such as amino acids, fatty acids, and sugars, are returned to the cytoplasm for metabolic recycling and energy homeostasis [13,14]. Functionally, the activation of autophagy serves as a critical cytoprotective mechanism by eliminating dysfunctional organelles and misfolded proteins, reducing oxidative stress, and limiting the accumulation of damage-associated molecular patterns (DAMPs) that promote inflammation. Through these actions, autophagy attenuates chronic inflammation, preserves genomic stability, and reduces carcinogenic potential. Collectively, the mTOR–ULK1–Beclin-1–LC3 signaling axis represents a fundamental homeostatic pathway that integrates metabolic sensing, stress adaptation, immune regulation, and tumor suppression [15]. In OSCC models, cannabinoids have been shown to induce autophagy-associated cell death by increasing reactive oxygen species (ROS) levels, upregulating Beclin-1, and increasing the accumulation of the autophagosomal marker microtubule-associated protein 1A/1B-light chain 3B (LC3B-II) [16]. In models of periodontitis and gingival inflammation, cannabinoids modulate the release of pro-inflammatory cytokines such as interleukin-6 (IL-6) and tumor necrosis factor-α (TNF-α), influence macrophage polarization toward a reparative phenotype, and promote the autophagic clearance of damaged organelle processes that collectively contribute to the resolution of inflammation and restoration of tissue homeostasis [11].

The therapeutic regulation of autophagy via cannabinoid pathways offers novel approaches to addressing oral diseases that often resist traditional treatments. Cannabinoids may enhance the regenerative potential of dental pulp stem cells by stimulating protective autophagy under oxidative stress [17]. Moreover, evidence suggests synergistic effects between cannabinoids and established therapeutics such as metformin, antibiotics, and chemotherapeutic agents, highlighting their potential as adjunctive therapies [18]. In cancer models, cannabinoids have been shown to enhance the cytotoxic efficacy of conventional chemotherapeutics (e.g., cisplatin, 5-fluorouracil, and doxorubicin) by promoting oxidative stress, inhibiting the PI3K/AKT/mTOR signaling pathway, and amplifying autophagy-mediated apoptosis [19,20]. Co-administration with antibiotics may potentiate antimicrobial efficacy and attenuate dysbiosis by modulating bacterial membrane integrity and host immune responses through cannabinoid signaling [21]. Importantly, their tissue-specific activity may enable targeted treatments with reduced systemic toxicity.

This review aims to provide a comprehensive examination of the molecular mechanisms through which cannabinoids modulate autophagy within the oral cavity, encompassing both physiological processes and pathological conditions. We discuss the expression and role of CB1 and CB2 receptors in oral tissues, evaluate in vitro and in vivo evidence for cannabinoid-autophagy interactions, and explore their translational implications in oral cancer, periodontitis, and dental tissue regeneration. By elucidating this promising therapeutic avenue, we aim to encourage further research and clinical investigation into cannabinoid-mediated modulation of autophagy as an innovative approach to oral healthcare.

## 2. Cannabinoid Signaling in Oral Tissues

The endocannabinoid system (ECS) is a complex lipid signaling network that governs various physiological activities, including inflammation, immune surveillance, pain modulation, and cellular homeostasis [22]. It consists of endogenous cannabinoids (endocannabinoids), their receptors (CB1 and CB2), and enzymes responsible for synthesis and breakdown [22]. Growing evidence indicates that cannabinoid signaling operates not only within the central nervous system but also throughout peripheral tissues, including the oral cavity, where both CB1 and CB2 are expressed in epithelial cells, fibroblasts, immune cells, dental pulp stem cells, and sensory neurons [23]. This widespread distribution underscores the potential of the ECS to influence oral inflammation, pain perception, tissue repair, and immune modulation.

### 2.1. Expression and Distribution of CB1 and CB2 in Oral Tissues

CB1 receptors are primarily expressed in the central and peripheral nervous systems, where they modulate neurotransmitter release and neural excitability. Activation of CB1 by THC mediates the characteristic psychoactive effects of cannabis, including alterations in perception, cognition, and motor coordination [24]. Nonetheless, CB1 is also expressed peripherally in oral tissues, including sensory neurons innervating the mucosa, gingival and lingual epithelial cells, and occasionally odontoblasts and dental pulp cells [25]. This peripheral expression enables CB1 to regulate sensory perception, inflammation, and epithelial barrier function in the oral environment.

CB2, initially thought to be restricted to immune cells, is now known to be widely expressed in various peripheral tissues, including bone, oral mucosa, and the nervous system. CB2 activation modulates inflammatory signaling pathways, regulates immune cell trafficking, and contributes to tissue repair and remodeling, underscoring its therapeutic relevance in chronic inflammatory and degenerative conditions [26]. In oral tissues, CB2 is abundantly expressed in macrophages, dendritic cells, neutrophils, and plasma cells. Elevated CB2 levels in inflamed gingiva suggest a role in chronic inflammation and immune polarization [27]. Additionally, CB2 is expressed in periodontal ligament fibroblasts and tooth pulp mesenchymal stem cells, where it plays a role in wound healing and regeneration [28]. Notably, both CB1 and CB2 activation affect the proliferation of OSCC cells, implicating cannabinoid signaling in cancer progression through mechanisms involving autophagy and oxidative stress [29].

### 2.2. Endogenous and Exogenous Cannabinoids in Oral Biology

Endocannabinoids, such as anandamide (AEA) and 2-arachidonoylglycerol (2-AG), are synthesized locally in the oral cavity and perform autocrine and paracrine functions [30]. These molecules are generated as needed in response to stimuli such as damage, inflammation, or infection, and interact with CB1 and CB2 receptors to facilitate downstream effects. Enzymes such as fatty acid amide hydrolase (FAAH) and monoacylglycerol lipase (MAGL) regulate the breakdown, thereby preserving homeostatic equilibrium [31]. Exogenous phytocannabinoids, primarily CBD and THC, interact with both CB1 and CB2 receptors and have been investigated for their therapeutic efficacy in oral health conditions [32]. CBD, a non-psychoactive phytocannabinoid, exhibits potent anti-inflammatory, antioxidant, and antiproliferative properties [33]. It functions as a negative allosteric modulator of the CB1 and can act as either an agonist or inverse agonist of the CB2, depending on tissue context and concentration [34]. In contrast, THC, a partial agonist at both CB1 and CB2 receptors, contributes to neuroprotective and anti-inflammatory effects through modulation of endocannabinoid signaling and downstream cytoprotective pathways [35].

### 2.3. Functional Outcomes of Cannabinoid Signaling in Oral Tissues

**Figure 1:**
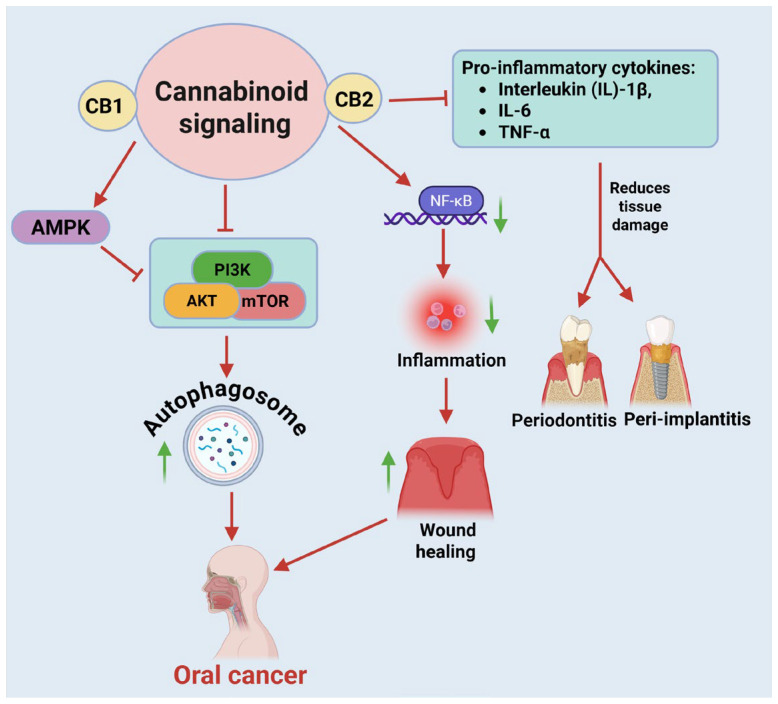
Cannabinoid-Mediated Regulation of Autophagy, Inflammation, and Tissue Repair in Oral Diseases. Activation of cannabinoid signaling stimulates AMP-activated protein kinase (AMPK) and suppresses the PI3K/AKT/mTOR pathway, leading to autophagosome formation and enhanced cellular clearance. Concurrently, CB2 receptor activation inhibits production of pro-inflammatory cytokines, including IL-1β, IL-6, and TNF-α, thereby contributing to the resolution of periodontitis and peri-implantitis. In parallel, CB2 receptor activation inhibits NF-κB signaling attenuation of inflammatory signaling limits tissue damage and promotes wound healing. Cannabinoid-induced autophagy and anti-inflammatory effects suppress oral cancer progression by disrupting tumor-supportive signaling pathways. Figure was created in BioRender by Rahman, M. (2025), Agreement number: FP28Z92637: https://BioRender.com.

Activation of the ECS within oral tissues can elicit a wide range of biological responses, including modulation of nociception, inflammatory signaling, oxidative stress, and cellular homeostasis. CB1 receptors located on peripheral sensory neurons and epithelial cells contribute to pain perception and neuromodulation, whereas CB2 receptors expressed on immune cells, fibroblasts, and endothelial cells play a predominant role in controlling inflammation and tissue remodeling (Figure 1). At the intracellular level, cannabinoid signaling converges on the canonical PI3K/AKT/mTOR–ULK1–Beclin-1–LC3 axis to regulate autophagic flux. Inhibition of mTOR and activation of AMPK collectively relieve suppression of ULK1, promoting Beclin-1–dependent autophagosome formation and LC3 lipidation. These coordinated events support cellular homeostasis, inflammatory resolution, and tissue repair in oral tissues. These molecular effects integrate to regulate cell survival, differentiation, and inflammatory resolution in oral tissues [4]. These pathways are essential for the breakdown of impaired proteins and organelles in epithelial and immune cells; for instance, in gingival and periodontal cells, cannabinoid-induced autophagy contributes to mitochondrial quality control and reduces proinflammatory cytokine release [36]. Additionally, activation of the CB2 receptor inhibits the production of pro-inflammatory cytokines, including Interleukin (IL)-1β, IL-6, and Tumor Necrosis Factor alpha (TNF-α), in gingival fibroblasts and macrophages [37]. This modulation reduces tissue damage in conditions like periodontitis and peri-implantitis. Cannabinoids also reduce oxidative stress and protect oral keratinocytes from ROS-induced apoptosis, which is critical in mitigating damage from smoking, alcohol, and environmental insults. By enhancing the activity of antioxidant enzymes such as superoxide dismutase (SOD) and catalase [38], and by stabilizing mitochondrial membrane potential, cannabinoids preserve cellular integrity under oxidative challenge. Moreover, CB2 receptor activation has been shown to attenuate NF-κB-mediated inflammatory signaling, thereby limiting downstream cytokine release and promoting epithelial resilience and wound healing in oral tissues [39]. In OSCC, CBD has been shown to exert potent cytotoxic and anti-metastatic effects [40]. demonstrated, using real-time electric cell–substrate impedance sensing, that CBD treatment significantly reduced OSCC cell viability, adhesion, and migratory capacity in a dose-dependent manner. These findings indicate that CBD disrupts tumor cell proliferation and metastatic behavior, potentially by modulating intracellular signaling pathways governing cell survival and motility [40]. The anticancer effects of cannabinoids in OSCC are attributed to the accumulation of reactive oxygen species (ROS), inhibition of AKT signaling, and downregulation of cyclin D1, leading to impaired cell proliferation and enhanced apoptotic activity. Beyond their tumor-suppressive role, cannabinoids also promote the viability and differentiation of dental pulp stem cells under conditions of oxidative and inflammatory stress, suggesting a dual capacity to both inhibit malignant transformation and support tissue regeneration within the oral environment [41]. Modulation of the cannabinoid receptor pathways, specifically CB1 and CB2, has been shown to facilitate reparative dentinogenesis and tissue regeneration in dental tissues. For example, cannabinoids such as CBD enhance migration, proliferation, and odonto/osteogenic differentiation of dental pulp stem cells (DPSCs), a key process in the formation of tertiary (reparative) dentin in response to tissue injury [42].

### 2.4. Interactions of Cannabinoid Receptors with Other Pathways

Cannabinoid signaling operates in close conjunction with several other regulatory systems, forming an integrated network that governs inflammation, metabolism, and tissue homeostasis. The ECS interacts extensively with the opioid, serotonergic, and dopaminergic systems, modulating nociception, mood, and stress responses through overlapping receptor signaling and shared second messengers. It connects with various pathways essential to oral tissue biology. The endocannabinoid system interacts with multiple pathways critical to oral tissue biology (Table 1). Opioid Pathway, CB1 and CB2 receptor crosstalk with μ-opioid receptors enhances analgesic effects, as cannabinoids and opioids can act synergistically to reduce nociceptive signaling in the oral mucosa and periodontal ligaments [43]. This modulation occurs through shared G-protein-coupled receptor (GPCR) mechanisms that decrease neurotransmitter release and dampen downstream excitatory signaling. Serotonin (5-HT) Pathway, CB1 receptors regulate serotonin release from sensory neurons, influencing mood, anxiety, and orofacial pain [44]. Additionally, CB2 activation can indirectly modulate serotonergic inflammation pathways, helping to control cytokine-mediated tissue damage [45]. Modulation of the MAPK/ERK pathways affects cell migration, survival, and differentiation, thereby maintaining tissue integrity, modulating inflammatory responses, and supporting homeostatic repair in oral tissues [46]. Cannabinoid receptor activation modulates this pathway in a context-dependent manner, often resulting in transient ERK phosphorylation that leads to growth arrest and differentiation rather than proliferation. Specifically, activation of CB1 or CB2 receptors can attenuate sustained ERK signaling through Gi/o-protein-coupled inhibition of adenylate cyclase and downstream cAMP-PKA pathways, ultimately suppressing oncogenic transcriptional programs such as c-Myc and cyclin D1. Simultaneously, cannabinoids exert an anti-inflammatory effect by suppressing the nuclear factor kappa-light-chain-enhancer of activated B cells (NF-κB) pathway. Cannabinoids downregulate the transcription of pro-inflammatory cytokines (e.g., TNF-α, IL-1β, IL-6) and adhesion molecules, thereby contributing to the resolution of inflammation and restoration of immunological homeostasis. This dual modulation/attenuation of MAPK/ERK and NF-κB signaling collectively reduces tumor cell invasiveness, oxidative stress, and inflammatory activation of the microenvironment, aligning with the observed cytostatic and cytoprotective effects of cannabinoid treatment in oral and other epithelial tissues [47].

### 2.5. Clinical Implications

Understanding the distribution and functional dynamics of cannabinoid receptors in oral tissues provides a compelling foundation for translating cannabinoid-based research into clinical practice. Cannabinoids, administered either topically (e.g., gels, rinses, or localized delivery systems) or systemically, have demonstrated potential in modulating inflammation, suppressing tumor proliferation, and enhancing tissue regeneration with a favorable safety profile [48]. Mechanistic studies, including investigations of synthetic cannabinoid analogs such as KM-233, further support this translational rationale by demonstrating that cannabinoid receptor activation can suppress the PI3K/AKT/mTOR and STAT3 survival pathways, induce mitochondrial depolarization, and trigger apoptosis in malignant cells [49]. Such findings suggest that cannabinoids can selectively target cancer cell metabolism and stress-response signaling while sparing normal tissue, an effect highly relevant to OSCC and other inflammatory oral pathologies.

Clinically, these mechanisms underscore the opportunity to design receptor-specific, dosage-controlled cannabinoid formulations that exploit these survival-to-death pathway shifts for therapeutic benefit. Topical or localized cannabinoid delivery systems could serve as adjuncts to conventional periodontal, mucosal, or oncologic therapies by restoring homeostatic autophagy and attenuating inflammatory cascades. However, realizing their full potential will depend on rigorously designed clinical trials that incorporate receptor selectivity, pharmacokinetic optimization, and patient-specific endocannabinoid profiling to define efficacy, safety, and individualized response patterns.

## 3. Autophagy Pathways Influenced by Cannabinoids

Autophagy, a lysosome-mediated catabolic mechanism, is essential for maintaining cellular homeostasis by degrading and recycling cytoplasmic components, especially under stress conditions such as food scarcity, oxidative stress, and infection [50]. In oral tissues, autophagy regulates inflammation, tissue remodeling, and cell survival, making it a key mechanism in conditions such as OSCC, gingivitis, and periodontitis. Cannabinoids influence autophagy primarily through CB1 and CB2 receptor signaling, modulating essential pathways that determine oral cell fate and the progression or resolution of oral diseases.

### 3.1. Core Autophagy Regulators Modulated by Cannabinoids

**Figure 2:**
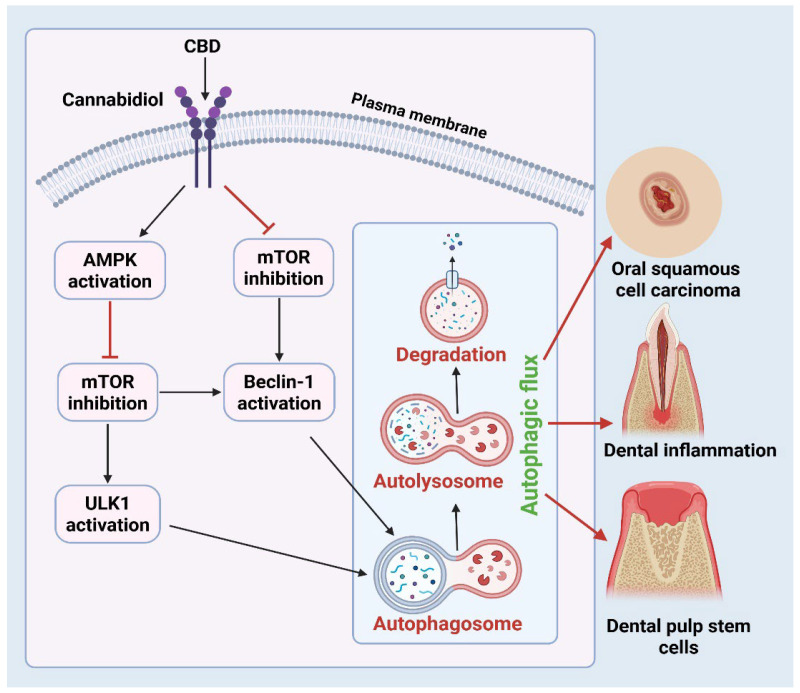
Mechanistic Overview of Cannabinoid-Induced Autophagy in Oral Cells. The molecular mechanism for the regulation of autophagy by cannabidiol (CBD), a representative phytocannabinoid, in oral cells. In response to a binding cannabinoid receptor on the cell plasma membrane, CBD stimulates the activation of AMP-activated protein kinase (AMPK) and concurrently inhibits the mechanistic target of rapamycin (mTOR), a critical negative regulator of autophagy. AMPK further inhibits mTOR and activates the Unc-51 Like Autophagy Activating Kinase 1 (ULK1), leading to autophagosome formation, while mTOR inhibition causes Beclin-1 activation, driving autophagosomal membrane nucleation. These events lead to autophagic flux progression, starting with autophagosome formation, autophagosome-lysosome fusion, forming autolysosomes, and ending with cytoplasmic degradation. Arrows indicate the proposed functional outcome of cannabinoid-triggered autophagy in three different oral diseases: (1) oral squamous cell carcinoma (OSCC) inhibition via autophagy-dependent cell death, (2) the resolution of dental inflammation through immunomodulation and clearance of damaged cellular components, and (3) the promotion of dental pulp stem cells (DPSCs) survival and differentiation under oxidative stress. The red block is inhibitory, while regular arrows show progressing pathway. Figure was created in BioRender by Rahman, M. (2025), Agreement number: FP28Z92637: https://BioRender.com.

As described in Section 1, cannabinoids regulate autophagy primarily through coordinated inhibition of PI3K/AKT/mTOR signaling and activation of the ULK1–Beclin-1–LC3 autophagic axis. In oral tissues, this core regulatory pathway is further modulated by energy-sensing mechanisms, inflammatory signaling, and cell-type–specific context. The increase in Beclin-1 expression due to cannabis exposure underscores its function in preparing cells for autophagic flux, especially in malignant tissues. Cannabinoids stimulate AMPK, an essential energy sensor that facilitates autophagy by inhibiting mTOR and directly phosphorylating ULK1 [51]. In oral epithelial and immunological cells, cannabinoid-induced activation of AMPK enhances autophagic flux, facilitating cellular clearance mechanisms [52]. This is especially pertinent in inflammation-related disorders, such as periodontitis, where the elimination of damaged organelles is crucial for sustaining cellular function (Figure 2). The AKT pathway, frequently stimulated in cancer and chronic inflammation, enhances cell survival and suppresses autophagy via mTOR activation [53]. Cannabinoids can inhibit AKT phosphorylation, hence diminishing its pro-survival signals and facilitating autophagy [54]. Persuasive evidence has shown that cannabis inhibits the PI3K/AKT/mTOR pathway in gingival fibroblasts, leading to the induction of autophagy and the cessation of inflammatory signals [55]. This action underscores the potential of cannabinoids to function as an anti-inflammatory compound through the control of autophagic mechanisms in periodontal disorders.

### 3.2. Oxidative Stress and Mitophagy

Cannabinoids also modulate autophagy via ROS, which act as signaling molecules that trigger mitophagy, the selective autophagic removal of damaged mitochondria [56]. Under normal physiological conditions, damaged or depolarized mitochondria are selectively removed by mitophagy to maintain cellular health. When a mitochondrion loses its membrane potential, PTEN-induced kinase 1 (PINK1) accumulates on the outer mitochondrial membrane. PINK1 then recruits and activates Parkin, an E3 ubiquitin ligase, which ubiquitinates various outer mitochondrial membrane proteins. This ubiquitination serves as a signal for autophagy adaptor proteins, such as p62, linking the mitochondrion to the forming autophagosome. The autophagosome subsequently fuses with a lysosome, where the damaged mitochondrion is degraded and its components recycled [57]. The buildup of ROS caused by cannabinoids results in mitochondrial depolarization, stability of PINK1 on the outer mitochondrial membrane, and the recruitment of Parkin [58]. This ROS-mediated process has a dual role: it enhances cell survival by eliminating defective mitochondria and mitigating oxidative damage, while also potentially inducing cell death in cancer cells when autophagy is excessive or unregulated. In oral cancer models, cannabinoid-induced reactive oxygen species production and mitophagy are associated with decreased tumor cell viability and increased chemosensitivity [59]. This signifies a potentially beneficial approach for adjuvant cannabis treatment in oral cancers.

### 3.3. Lysosomal Biogenesis and Autophagic Dynamics

Cannabinoids appear to influence the final stages of autophagy, encompassing lysosomal biogenesis and the fusion of autophagosomes with lysosomes. Transcription factor EB (TFEB), a major regulator of lysosomal genes, is activated in response to cannabinoid-induced inhibition of mTOR [54]. Upon translocation to the nucleus, TFEB augments the transcription of genes associated with lysosome biogenesis and autophagic degradation, hence facilitating comprehensive autophagic flux [60]. This system is essential in oral tissues for the efficient removal of inflammatory debris, bacterial byproducts, and cellular waste. Improved lysosomal turnover in gingival tissues promotes enhanced immunological homeostasis and periodontal integrity.

### 3.4. Contextual Function of Autophagy in Oral Pathologies

The biological effects of cannabinoid-induced autophagy in oral tissues are significantly context-dependent. In oral squamous cell carcinoma, autophagy may promote programmed cell death or enhance cellular sensitivity to chemotherapeutics [61]. In contrast, in non-malignant tissues, autophagy serves as a preventive mechanism against chronic stress, infection, and oxidative damage [62]. For instance, in OSCC, cannabinoids enhance the expression of LC3B-II and Beclin-1, induce G1/S phase arrest, and facilitate autophagic cell death [54]. Cannabinoids stimulate the removal of inflammatory debris, promote macrophage polarization towards anti-inflammatory phenotypes, and diminish pro-inflammatory cytokine production in gingivitis and periodontitis [48]. Cannabinoids enhance the survival and functionality of stem cells in dental pulp regeneration via autophagy-mediated cytoprotection [63].

## 4. Therapeutic Implications in Oral Diseases

### 4.1. Oral Cancer

**Figure 3:**
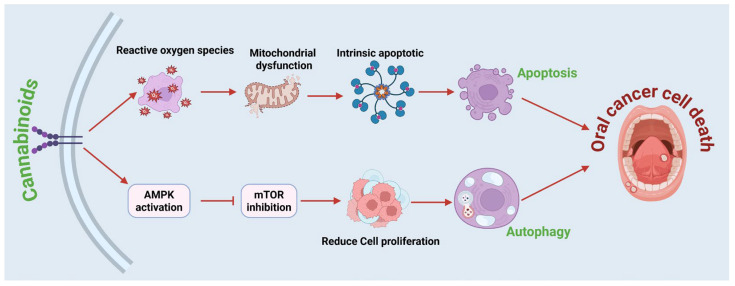
Role of cannabinoids in induction of autophagy and apoptosis in oral cells. The dual mechanism by which cannabinoids induce autophagy and apoptosis, leading to the death of oral cells. When cannabinoids bind to cannabinoid receptors on the cell membrane, they activate AMPK signaling and inhibit mTOR, resulting in decreased proliferation of oral cancer cells and increased autophagy. Cannabinoids also increase ROS production, leading to mitochondrial dysfunction and activation of the intrinsic apoptotic pathway. These processes increase autophagic flux and apoptotic signaling, leading to the degradation of cellular components and the effective elimination of cancer cells. Figure was created in BioRender by Rahman, M. (2025), Agreement number: FP28Z92637: https://BioRender.com.

The interaction between cannabinoid signaling and autophagy holds promising therapeutic potential for managing oral diseases, especially OSCC, which can be an aggressive malignancy with a poor prognosis in advanced stages. Cannabinoids can induce both apoptosis and autophagy, thereby disrupting tumor progression. Apoptosis is often triggered by cannabinoid-induced ROS generation, leading to mitochondrial dysfunction and activation of intrinsic apoptotic pathways. In parallel, cannabinoids modulate the AMPK/mTOR axis, in which AMPK acts as a cellular energy sensor and suppresses mTOR signaling, leading to reduced tumor cell proliferation and enhanced autophagy [64,65]. Low doses of cannabinoids were found to be effective in inhibiting the proliferation of oral cancer cells [65]. Further, excessive autophagy activation can lead to cancer cell death, and cannabinoids significantly increase LC3B-II and Beclin-1 levels, markers of autophagic activation [66,67]. This process enhances the clearance of dysfunctional mitochondria and protein aggregates through lysosomal degradation [68] (Figure 3). Targeting the G1/S checkpoint is a critical therapeutic strategy in OSCC, as dysregulation of cyclin D1 and CDK4/6 promotes unchecked progression into the S phase and drives malignant proliferation [69]. In normal physiology, this checkpoint safeguards genomic integrity by halting the cycle to allow DNA repair or by initiating programmed cell death when damage is irreparable. In OSCC, sustained Rb phosphorylation and E2F activation bypass these controls, facilitating tumor growth and treatment resistance. Agents that restore G1/S arrest can suppress proliferation and redirect cells toward apoptosis or autophagy depending on intracellular stress cues. Cannabinoids such as CBD and THC have been shown to downregulate cyclin D1, inhibit CDK4/6, and reduce PI3K/AKT/mTOR signaling, collectively leading to G1-phase accumulation and enhanced autophagic and apoptotic activity. These findings suggest that cannabinoids may help re-establish checkpoint control, attenuate tumor progression, and offer adjunctive benefit in the management of OSCC [70,71]. It is important to emphasize that current human evidence supporting cannabinoid use in oral cancer remains preliminary and largely extrapolated from preclinical models, and no randomized clinical trials have yet demonstrated oncologic efficacy in OSCC.

### 4.2. Periodontitis and Gingival Inflammation

Chronic inflammation in periodontitis and gingivitis reflects an imbalance between pro-inflammatory and anti-inflammatory signaling, driven by elevated pro-inflammatory cytokines and chemokines, such as IL-6, TNF-α, and IL-1β [72]. Given this central role of inflammation, the ECS, particularly CB2 receptor signaling, represents an attractive therapeutic target. In vitro, the selective CB2 receptor agonist SMM-189 significantly attenuated the expression of pro-inflammatory markers in primary human periodontal ligament fibroblasts [73]. Clinical evidence further supports cannabinoid-based interventions: a CBD-containing mouthwash improved plaque and gingival indices while modulating microbial composition, and a CBD dental gel reduced periodontal inflammation and inhibited the growth of pathogenic bacteria without adverse effects [74,75]. Kiełbratowski and colleagues randomized 40 adults with gingivitis into 4 groups, including a group receiving mouthwash containing CBD and spilanthol. After 42 days of twice-daily use, the CBD-containing mouthrinse group showed a significant reduction in bleeding on probing, a decreased plaque index, and anti-inflammatory effects [75]. Jirasek and colleagues conducted a 3-arm randomized trial with 90 adults with periodontitis (stages I–IV) treated with 1% CBD toothpaste for 56 days in people with periodontitis. The results showed a significant improvement in gingivitis and gum bleeding, with no reported side effects [74]. Umpreecha and colleagues conducted a randomized clinical trial that demonstrated that topical 0.1% CBD is effective for recurrent oral ulcers. After treatment, they found significantly reduced ulcer size, accelerated ulcer healing, and effective pain relief, with no reported side effects [76]. In addition, our recent exploratory feasibility study, the Simple Tooth Extraction with Analgesic Phytocannabinoid (SWAP) pilot trial, evaluated oral cannabidiol (CBD) for the management of acute post-extraction pain. This four-arm randomized pilot study (*n* = 8) compared two oral CBD concentrations (17 mg/mL and 37 mg/mL) with placebo and treatment-as-usual (ibuprofen/acetaminophen). Pain intensity was assessed using ecological momentary assessment over the first 72 h following extraction. Although the study was not powered to assess efficacy and baseline imbalances were observed, participants receiving the higher-concentration CBD (37 mg/mL) demonstrated a pain trajectory qualitatively like those observed with standard non-opioid therapy, whereas the lower-concentration CBD and placebo groups showed limited analgesic effect. No safety concerns were identified, and participant adherence exceeded 75%. These findings are presented as descriptive and hypothesis-generating, supporting feasibility rather than clinical effectiveness [77]. Although these early human studies suggest potential anti-inflammatory and analgesic effects, they are exploratory in nature, limited by small sample sizes and short follow-up durations, and should be interpreted as hypothesis-generating rather than definitive clinical evidence. These findings highlight the dual antimicrobial and immunomodulatory actions of cannabinoids. By attenuating pro-inflammatory cytokine production and promoting autophagic clearance of damaged cellular components, cannabinoids may help preserve tissue integrity and improve healing outcomes in periodontal disease. Such properties highlight the potential role of ECS-targeted therapeutics in managing chronic oral inflammation.

### 4.3. Dental Pulp and Regeneration

**Figure 4:**
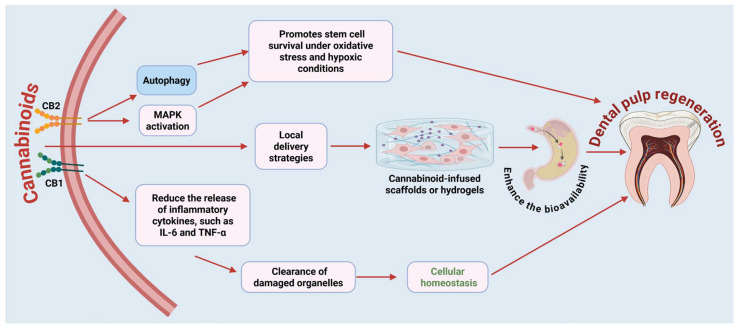
Proposed molecular processes responsible for dental pulp regeneration via cannabinoid receptor activation. Cannabinoids interact with CB1 and CB2 receptors expressed on human dental pulp cells. Activation of CB2 receptors leads to the activation of MAPK signaling and induction of autophagy. These processes contribute to stem cell survival under conditions of oxidative stress and hypoxia, which are characteristic of injured or inflamed pulp tissues. In parallel, cannabinoid signaling decreases the release of pro-inflammatory cytokines such as IL-6 and TNF-α, thereby enhancing the clearance of damaged organelles and restoring cellular homeostasis. Local delivery systems such as cannabinoid-infused scaffolds or hydrogels are being investigated to enhance targeted delivery and bioavailability within the confined space of the pulp chamber. These biomaterials not only provide localized delivery but also support the regeneration of the dentin-pulp complex by promoting angiogenesis, neurogenesis, and tissue remodeling. Figure was created in BioRender by Rahman, M. (2025), Agreement number: FP28Z92637: https://BioRender.com.

Cannabinoids also demonstrate regenerative potential in dental pulp biology. Activation of CB1 and CB2 receptors in human dental pulp cells has been shown to modulate cell proliferation, migration, and differentiation during bone/dentin regeneration [78]. These effects are primarily mediated by CB2-dependent MAPK signaling and autophagy pathways [79]. Autophagy induction, as evidenced by increased expression of LC3B-II and Beclin-1, promotes stem cell survival under oxidative and hypoxic conditions, conditions frequently encountered in inflamed or injured pulp tissue. Additionally, cannabinoid signaling may reduce the release of inflammatory cytokines, such as IL-6 and TNF-α, while promoting the clearance of damaged organelles to maintain cellular homeostasis. Local delivery strategies, such as cannabinoid-infused scaffolds or hydrogels, are being investigated to enhance the bioavailability of these substances within the restricted pulp chamber [80,81,82]. These approaches could complement existing regenerative endodontic protocols by supporting angiogenesis, neurogenesis, and the restoration of the dentin-pulp complex (Figure 4). However, it is essential to consider dose-dependent effects, receptor-specific outcomes, and regulatory restrictions for successful clinical translation.

## 5. Mechanistic Insights and Molecular Targets

Recent studies highlight the potential of cannabinoids to modulate autophagy via multiple pathways, making them promising treatments for OSCC, gingivitis, and nerve-related oral conditions. Central to this process is the mTOR/AKT/PI3K signaling axis, a major regulator of cell growth, survival, motility, and metabolism in oral cancer. CBD primarily inhibits this pathway, which in turn activates autophagy [64]. In cases of OSCC, this activation reduces tumor cell viability, suggesting a cytotoxic mechanism. Additionally, CBD’s anti-inflammatory properties support its application in treating gingival disease, where autophagy plays a crucial role in maintaining immune balance. THC induces autophagy primarily by activating CB1/CB2 receptors, which can increase ROS and trigger endoplasmic reticulum (ER) stress. This process leads to the release of Beclin-1 from its inhibitory complex with BCL-2, facilitating the formation of autophagosomes and increasing LC3-II levels. The ROS-dependent autophagy induced by THC has been shown to have cytotoxic effects on oral carcinoma cells [70]. These findings indicate that THC could be a promising candidate for inducing autophagic cell death in oral tumors [83]. However, excessive ROS can lead to autophagy-dependent apoptosis or promote carcinogenesis when autophagy is insufficient [84]. Important considerations and challenges are associated with THC use due to its treatment dose and psychoactive effects [85]. Low doses of THC can promote cell survival, while higher doses may induce cell death. Therefore, precise dosing is critical to avoid unintended pro-survival autophagy.

The synthetic cannabinoid WIN 55,212-2 is a full agonist that primarily acts by activating the CB1 receptor and promoting Beclin-1-dependent autophagy. This mechanism has been linked to modulating nerve-inflammation and neuropathic pain in oral tissues. Functional studies often involve pharmacological inhibition of the CB1 receptor using antagonists, such as rimonabant, or employing genetic knockdown techniques to confirm receptor-specific effects [86]. The role of autophagy is further confirmed by silencing Beclin-1 and examining LC3-II dynamics. The anti-inflammatory effects are typically assessed by measuring pro-inflammatory cytokine levels and conducting neuronal survival assays. Thus, WIN 55,212-2 presents a promising option for alleviating oral neuropathic conditions through cannabinoid-mediated autophagy pathways [87] (Table 2).

## 6. Challenges and Future Perspectives

Despite promising mechanisms, several challenges hinder the clinical application of cannabinoid-mediated autophagy for oral-related diseases. A critical consideration is the dose-dependent biphasic response of cannabinoids [90,91]. At low concentrations, cannabinoids may exert cytoprotective and anti-inflammatory effects, supporting cell survival and tissue repair [92]. In contrast, higher concentrations can trigger pro-apoptotic signaling and excessive autophagy, potentially leading to cytotoxicity in healthy oral tissues. Closely related to dose sensitivity is the highly context-dependent role of autophagy, particularly in oral squamous cell carcinoma (OSCC). As comprehensively reviewed by Anderson and O’Sullivan, autophagy can exert tumor-suppressive functions in early carcinogenesis by maintaining genomic stability, limiting oxidative and metabolic stress, and facilitating the clearance of damaged organelles and proteins. In contrast, in established OSCC, autophagy frequently acts as a pro-survival adaptive mechanism, enabling tumor cells to endure hypoxia, nutrient deprivation, and therapeutic stress, thereby contributing to tumor progression, immune evasion, and resistance to chemotherapy and radiotherapy [93]. Consistent with this paradigm, Zeng and colleagues highlight that while modulation of autophagy may enhance the efficacy of chemotherapy, radiotherapy, and immunotherapy in OSCC, such benefits are highly conditional and dependent on precise temporal and quantitative control of autophagic flux [94]. Potential therapeutic effects are mediated through pathways including mTOR and AMPK signaling, alterations in tumor metabolism, and reshaping of the tumor immune microenvironment. However, inappropriate or sustained activation of autophagy may paradoxically promote tumor cell survival and treatment resistance. Importantly, the therapeutic impact of autophagy modulation in OSCC is influenced by multiple variables, including tumor stage, timing of intervention, p53 status, immune contexture, and microenvironmental stressors. These observations underscore that autophagy cannot be viewed as a uniformly beneficial target in OSCC. Instead, it represents a biologically dual, context-sensitive process that requires carefully stratified, context-specific approaches. Accordingly, biomarker-guided patient selection and targeted delivery strategies will be essential to safely harness autophagy modulation while minimizing pro-tumorigenic effects. A further complication is receptor specificity, as the functions of CB1 and CB2 receptors often overlap and vary across cell types, pathological states, and tissue distributions, making selective therapeutic targeting difficult. This receptor overlap complicates the development of highly selective cannabinoid-based therapeutics. Moreover, limitations in bioavailability and delivery represent significant obstacles to clinical translation. Cannabinoids are highly lipophilic, leading to poor water solubility, variable absorption, and extensive first-pass metabolism, all of which reduce systemic availability and result in unpredictable plasma concentrations. These pharmacokinetic properties complicate dose standardization and contribute to interpatient variability in therapeutic response. Oral mucosal tissues create barriers to cannabinoid penetration, and systemic delivery often results in low local concentrations. Therefore, developing localized delivery systems, such as cannabinoid-infused dental resins, gels, or nanoparticle carriers, is essential for achieving therapeutic levels at the disease site. In addition, regulatory constraints pose significant barriers. The inconsistent global legal status of cannabis-derived therapeutics, coupled with variations in purity standards and manufacturing requirements, hinders large-scale clinical trials and translation into dental practice.

While cannabinoids have been widely investigated for their anti-inflammatory, analgesic, and potential antitumor properties, increasing evidence indicates that cannabinoid exposure may also pose biological risks, particularly in the context of oral and head and neck cancers. These risks are highly context-dependent and influenced by dose, cannabinoid composition, route of administration, receptor engagement, and disease stage. THC, the primary psychoactive component of cannabis, has been shown in experimental models to induce oxidative stress and DNA damage, including increased ROS production, chromosomal aberrations, and impaired DNA repair mechanisms. In vitro and in vivo studies demonstrate that marijuana smoke and THC exposure can cause measurable DNA strand breaks and genomic instability, raising concerns about carcinogenic potential, particularly in tissues with direct exposure, such as the oral epithelium [95,96,97]. Route of administration is an additional clinically relevant determinant of risk. Smoked cannabis contains many of the same carcinogenic combustion products found in tobacco smoke, including polycyclic aromatic hydrocarbons and nitrosamines. Epidemiologic studies have linked chronic cannabis smoking with increased risk of head and neck malignancies, including cancers of the oral cavity, pharynx, and larynx, although findings vary by exposure intensity and confounding tobacco use [98,99]. These risks are largely attributable to combustion-derived toxicants rather than cannabinoid receptor–mediated signaling. Another important consideration is the role of cannabinoid-induced autophagy, which has frequently been proposed as a mechanism underlying cannabinoid-associated tumor suppression. However, substantial oncologic literature indicates that autophagy can also function as a cytoprotective survival mechanism, enabling tumor cells to withstand metabolic stress, hypoxia, and cytotoxic therapies [100]. In certain contexts, autophagy induction may therefore promote tumor persistence, progression, or therapeutic resistance rather than tumor cell death [101]. Experimental evidence suggests that inhibition of autophagy can enhance chemosensitivity and limit cancer cell survival, underscoring the dual and context-dependent nature of this pathway [101]. Collectively, these findings highlight that the biological effects of cannabinoids in oral cancer are not uniformly antitumorigenic. Instead, outcomes depend on multiple variables, including cannabinoid subtype (e.g., THC versus CBD), concentration, receptor engagement (CB1, CB2, and non-canonical receptors), cellular context, and timing of exposure. Accordingly, cannabinoid-induced autophagy and cytoprotective signaling should be interpreted cautiously, particularly when extrapolating preclinical findings to clinical oncology. A comprehensive understanding of these risks is essential for guiding future preclinical and clinical investigations. Well-designed studies incorporating dose–response analyses, mechanistic biomarkers, and clinically relevant oral cancer models are required to delineate conditions under which cannabinoids may confer therapeutic benefit versus harm. In contemporary dental practice, postoperative pain and inflammation are routinely managed with established non-opioid regimens, including non-steroidal anti-inflammatory drugs (NSAIDs), acetaminophen, and, in selected cases, short courses of corticosteroids, which together constitute the standard of care following procedures such as simple tooth extractions. In periodontal disease and oral squamous cell carcinoma (OSCC), established adjunctive therapies, including mechanical debridement, antimicrobial strategies, radiotherapy, chemotherapy, immunotherapy, and targeted molecular agents, remain the cornerstone of disease management. Against this backdrop, cannabinoids should be evaluated relative to, rather than in isolation from, these existing therapeutic standards.

To provide preliminary comparative context, we incorporated findings from our exploratory Simple Tooth Extraction with Analgesic Phytocannabinoid (SWAP) pilot trial, which directly compared oral CBD with standard non-opioid therapy (ibuprofen/acetaminophen) following simple dental extractions [77]. In this four-arm randomized feasibility study (*n* = 8), two CBD concentrations (17 mg/mL and 37 mg/mL) were evaluated alongside placebo and treatment-as-usual. Pain intensity was captured using ecological momentary assessment over the first 72 h post-extraction. While the study was not powered to assess efficacy, baseline imbalances limited quantitative interpretation. Participants receiving higher-concentration CBD (37 mg/mL) demonstrated pain trajectories qualitatively similar to those observed with standard ibuprofen/acetaminophen therapy, whereas lower-dose CBD and placebo showed limited analgesic effects. These findings are presented as descriptive and hypothesis-generating, emphasizing feasibility rather than clinical equivalence or superiority, and do not support replacing established NSAID-based or corticosteroid regimens with cannabinoids. Rather, it suggests that CBD may warrant further investigation as a potential adjunct or alternative for selecting patients, such as those with contraindications or intolerance to NSAIDs, provided that safety, dosing, and efficacy are confirmed in larger trials. In the context of periodontal disease and OSCC, current evidence remains even more preliminary. Cannabinoids have not been shown to outperform standard adjunctive therapies, and their role, if any, is likely to be supportive rather than disease-modifying. Collectively, these observations underscore the necessity of head-to-head, adequately powered randomized trials comparing cannabinoids directly with standard anti-inflammatory agents and established oncologic adjunct therapies. Such studies should incorporate standardized dosing, mechanistic biomarkers, and clinically meaningful outcomes to accurately define the therapeutic niche, if any, of cannabinoids within dental and oral oncology care.

Future research should focus on finding cannabinoid analogs with high selectivity for oral tissues, developing sustained-release local delivery systems, and integrating personalized medicine approaches to tailor cannabinoid-based autophagy modulation to individual patient profiles. Large-scale, randomized controlled trials are crucial for confirming the efficacy, safety, and long-term outcomes across diverse oral disease models. Therefore, future research should prioritize the development of non-psychoactive cannabinoid derivatives, such as CB2-selective agonists, to induce harmless autophagy for the management of oral diseases without side effects. Combining cannabinoids with autophagy modulators, such as mTOR inhibitors, could enhance efficacy in oral cancer treatment, potentially offering an alternative to chemotherapy.

## 7. Conclusions

Cannabinoid-based treatments show promise for managing oral diseases by controlling inflammation and promoting tissue regeneration through specific pathways. However, challenges such as receptor specificity, dose-dependent effects, and limited bioavailability impede their clinical use. Importantly, both the therapeutic potential and the biological risks of cannabinoid exposure, including THC-associated DNA damage, carcinogenic combustion products, and the possibility of cytoprotective autophagy in malignant cells, must be carefully weighed when considering clinical translation. Accordingly, future research should prioritize targeted delivery systems and rigorously designed clinical trials to mitigate these risks and to critically evaluate the safety and efficacy of cannabinoids, thereby determining whether they can be responsibly advanced as viable therapies for oral health.

## Figures and Tables

**Table 1:** Overview of Cannabinoid Signaling Interactions in Oral Tissues.

Pathway	Cannabinoid Interaction	Main Effect in Oral Tissues	Reference
Opioid Pathway	CB1/CB2 interact with μ-opioid receptors via shared GPCR signaling	Analgesia; reduced oral and periodontal pain	[43]
Serotonin (5-HT) Pathway	CB1 regulates serotonin release; CB2 modulates inflammatory serotonin signaling	Modulation of mood, anxiety, orofacial pain; reduced inflammation	[44,45]
MAPK/ERK Pathway	CB1/CB2 attenuate sustained ERK activation	Growth arrest, differentiation, tissue repair	[46]
NF-κB Pathway	CB1/CB2 suppress NF-κB–mediated transcription	Decreased pro-inflammatory cytokines; reduced oxidative stress	[47]

**Table 2:** Mechanistic Insights and Molecular Targets.

Cannabinoid	Autophagy Target	Effect	Disease Context	Reference
CBD	mTOR, AKT	Inhibition → autophagy activation	OSCC, gingivitis	[64,88,89]
THC	ROS, LC3-II	ROS-induced autophagy	Oral carcinoma	[70,83,84]
WIN 55,212-2	CB1-R, Beclin-1	Induction of CB1-mediated autophagy	Nerve-associated inflammation	[86,87]

## Data Availability

No new data were created or analyzed in this study. Data sharing is not applicable to this article.
